# Boosting third-harmonic generation by a mirror-enhanced anapole resonator

**DOI:** 10.1038/s41377-018-0051-8

**Published:** 2018-07-25

**Authors:** Lei Xu, Mohsen Rahmani, Khosro Zangeneh Kamali, Aristeidis Lamprianidis, Lavinia Ghirardini, Jürgen Sautter, Rocio Camacho-Morales, Haitao Chen, Matthew Parry, Isabelle Staude, Guoquan Zhang, Dragomir Neshev, Andrey E. Miroshnichenko

**Affiliations:** 1 School of Engineering and Information Technology, University of New South Wales, Canberra, ACT 2600 Australia; 2 Nonlinear Physics Centre, The Australian National University, Canberra, ACT 2601 Australia; 3 Department of Physics, Politecnico di Milano, Piazza Leonardo Da Vinci 32, 20133 Milan, Italy; 4 Institute of Applied Physics, Abbe Center of Photonics, Friedrich Schiller University Jena, Albert-Einstein-Str. 15, 07745 Jena, Germany; 5 The MOE Key Laboratory of Weak Light Nonlinear Photonics, School of Physics and TEDA Applied Physics Institute, Nankai University, Tianjin, 300457 China

## Abstract

We demonstrate that a dielectric anapole resonator on a metallic mirror can enhance the third harmonic emission by two orders of magnitude compared to a typical anapole resonator on an insulator substrate. By employing a gold mirror under a silicon nanodisk, we introduce a novel characteristic of the anapole mode through the spatial overlap of resonantly excited Cartesian electric and toroidal dipole modes. This is a remarkable improvement on the early demonstrations of the anapole mode in which the electric and toroidal modes interfere off-resonantly. Therefore, our system produces a significant near-field enhancement, facilitating the nonlinear process. Moreover, the mirror surface boosts the nonlinear emission via the free-charge oscillations within the interface, equivalent to producing a mirror image of the nonlinear source and the pump beneath the interface. We found that these improvements result in an extremely high experimentally obtained efficiency of 0.01%.

## Dielectric nanophotonics: mirror-enhancement

The use of a thin metallic substrate can dramatically enhance the magnitude of third harmonic generation (THG) that is possible from a silicon nanodisk anapole resonator. Lei Xu and coworkers from Australia, Italy, Germany and China fabricated 200nm-thick amorphous silicon nanodisks on a gold film. As with other dielectric nanostructures, the silicon nanodisk supports nonradiating anapole states that result from the destructive interference of electric and toroidal dipole moments in the far-field. At the same time, such anapole states provide near-field enhancement inside the silicon nanodisk and can strengthen nonlinear effects such as THG. Xu and coworkers have now found that the THG can be further boosted by another two orders of magnitude to reach a conversion efficiency of 0.01% by using a gold film substrate to generate a virtual mirror image that coherently amplifies the structure’s nonlinear response.

## Introduction

The field of nanophotonics, which aims for the efficient control of light at the nanoscale dimensions, has experienced significant growth recently due to its wide variety of applications, ranging from fully functional photonic circuitry to advanced metadevices^1,2^. Among all branches of nanophotonics, nonlinear nanophotonics^3^, e.g., frequency conversion, wave mixing, and all-optical switching, is a rapidly developing research field benefiting applications such as chip-based light sources^4^, nanolasers^5,6^, optoelectronic devices^7^, imaging^8^, and sensing^9^. During the last decade, nanoplasmonics has played a key role in achieving a strong nonlinear response, which arises from coherent oscillations of conduction electrons near the surface of plasmonic nanostructures^10–17^. However, the performance of plasmonics has been bottlenecked by high Ohmic losses, small mode volumes, and low laser damage threshold. More recently, all-dielectric nanoparticles with low optical losses have been considered as an alternative to overcome the current limitations of plasmonic nanostructures^18,19^.

Dielectric nanostructures offer an attractive platform for various nonlinear effects due to their ability to efficiently confine and manipulate light at the nanoscale, based on the control of both optically induced electric and magnetic Mie type resonances^19–21^. Nanostructures made of high-index semiconductors with a strong nonlinear response, i.e., third order susceptibility for materials such as Si, Ge, as well as second order susceptibility for materials such as AlGaAs, GaAs, and GaP, have been widely explored for nonlinear effects at the nanoscale. It has been demonstrated that the nonlinear responses in such nanostructures can be substantially enhanced^22–29^. Moreover, complex nanostructures, such as hybrid plasmonic/dielectric nanoparticles^14,30^ and Fano-resonant dielectric metasurfaces^31,32^, have been exploited to further boost the nonlinear effects. Recently, it was demonstrated that high-index dielectric nanoparticles can support nonradiating anapole excitations^33^. Anapole modes originate from the overlap of co-excited electric dipole (ED) and toroidal dipole (TD) moments that have the same scattering magnitude but are out of phase. This results in the complete cancellation of their scattering contributions in the far-field while exhibiting strong near-field enhancement inside the nanoparticle. Thus, excitation of such nonradiative anapole modes can significantly improve the nonlinear conversion efficiency^30,34^.

Enhanced third-harmonic generation (THG) has previously been demonstrated in thin Ge nanodisks through the excitation of anapoles^34^. For a single nanodisk configuration, the anapole mode is formed by the spectral overlap between the off- resonantly excited electric and toroidal dipole modes resulting in destructive far-field interference. Recently, it has been shown that the anapole mode supported by a Si nanodisk can be boosted by a plasmonic resonance from the surrounding metal nanostructures, producing high electric near-field enhancement within the Si nanostructure and thus significantly improving the third-harmonic (TH) conversion efficiency^30^.

Here, we present a novel approach for the incorporation of an anapole excitation to further boost the THG efficiency. We have designed and fabricated dielectric nanodisks on top of a Au film in order to engineer and control the Mie resonances for both linear and nonlinear processes. The Au film acts as a mirror that generates a virtual image that coherently amplifies the nonlinear response. The effects of metallic substrates on metallic nanoparticles have been previously studied theoretically^35–37^ and experimentally^38–41^ by employing enhanced near-field localization in the middle gap between them. This has led to a number of applications, such as scanning near-field optical microscopy^42^, surface-enhanced Raman scattering^43^, plasmon-based biosensors^44^, and optical tweezers^45^. However, the effect of a metallic film on Mie resonators based on all-dielectric nanoparticles still requires further exploration.

We have demonstrated that the benefits of this approach are two-fold: first, Cartesian electric dipole and toroidal dipole modes are both resonantly excited with the same scattering magnitude and spatially overlap with the help of the enhanced magnetic field at the interface. This enhancement occurs because of the free electron oscillations and associated couplings from the metal film. This is a significant advantage over the previously reported anapole excitations in which the electric and toroidal dipole modes generally overlap close to the minima of both modes^33,34^. Thus, we achieved a significantly enhanced localized electric field inside the disk via the anapole mode associated with a magnetic quadrupole mode. Second, a mirror image of the nonlinear source is created by the metal film during the nonlinear process. These advantages result in an experimentally obtained total TH conversion efficiency of 0.01%, which, to the best of our knowledge, is the highest efficiency reported to date for THG in nanostructures.

## Results

**Fig. 1:**
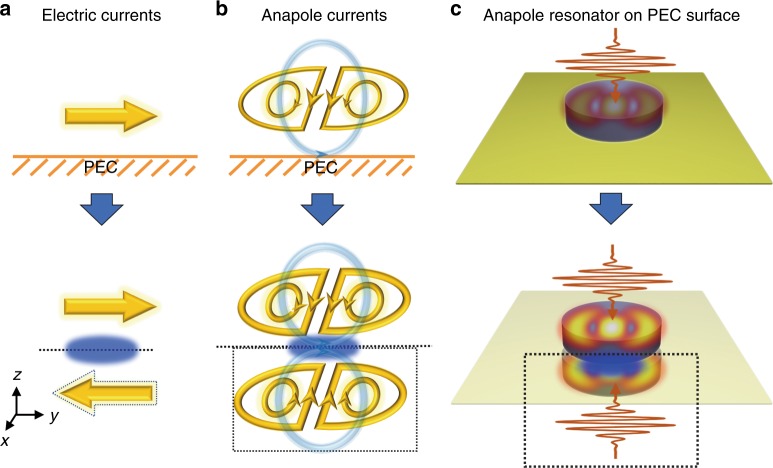
Schematic illustration of currents excited in resonator-on-PEC-surface systems. **a** An electric current on a PEC substrate. **b**, **c** Illustrate the current and field distributions of an anapole resonator on a PEC substrate, respectively

We first demonstrate the effect of an electric mirror on electric currents. With an electric current placed near a perfect electric conductor (PEC) surface, the excited free electron oscillations in the adjacent PEC surface will further affect the near-field and far-field properties of such systems^46–48^. Such an effect can be considered to be an oppositely oriented image of the electric current by using the image dipole model^40,49–51^ as shown in Fig. 1a. The magnetic field near the surface will be enhanced (a magnetic hotspot will be formed near the mirror surface) by these currents due to the coupling of the currents and the free electron oscillations within the PEC surface^52^. Indeed, the PEC surface provides the ability to engineer both the current distributions and the field distributions by inducing image counterparts. Next, we consider a configuration with an excited anapole mode supported by a high-index dielectric nanodisk under plane wave irradiation^33^. The PEC surface effect will result in a configuration equivalent to that obtained with two counter-propagating beams with the same intensity but out of phase that are incident on two anapole resonators. As a result, a magnetic hotspot will be formed near the interface. This magnetic hotspot will further boost the excitation of the electric dipole and toroidal dipole modes by enhancing the corresponding magnetic circular current components, as shown in Fig. 1b, c. In such a case, it is expected that a strongly excited anapole mode will be obtained due to the overlap between the strongly excited Cartesian electric and toroidal dipole modes, and the near-field distribution of the resonator can therefore be significantly magnified.

**Fig. 2:**
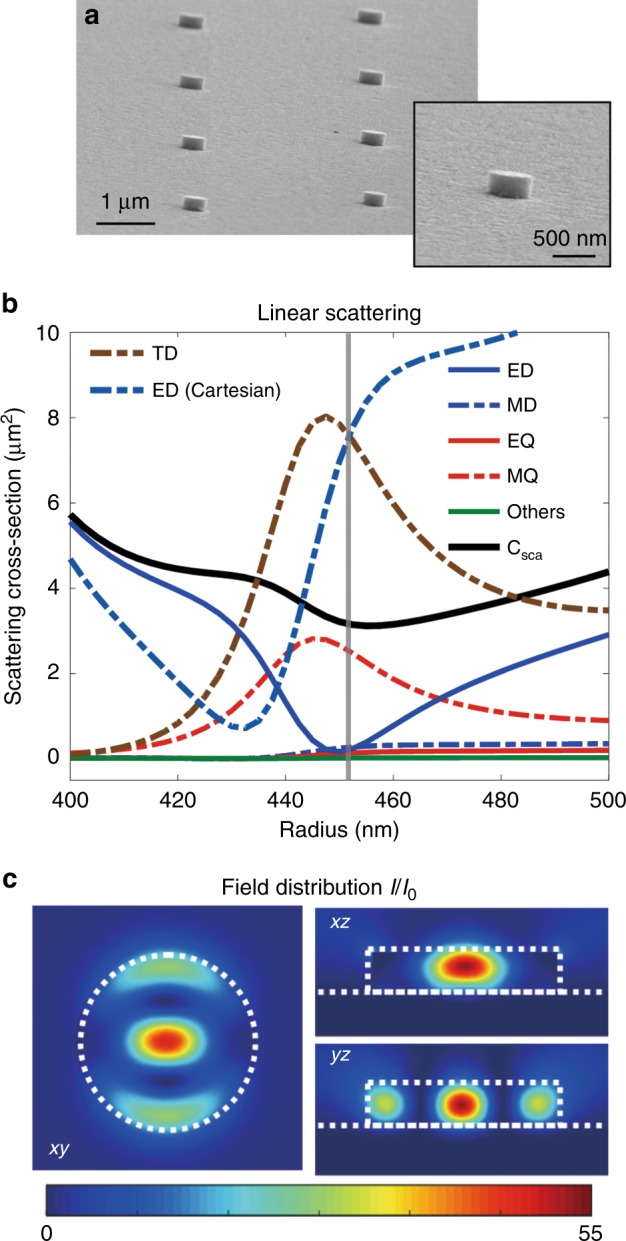
**a** SEM image of the fabricated ROM configuration. **b** Calculated linear scattering and multipolar decom- position, under plane wave excitation, for the ROM nanostructure with different disk radii. **c** The near-field profiles for the ROM configuration with disk radius *r* = 450 nm

In our experiments, we fabricated amorphous silicon nanodisks with a height of 200 on a 200 nm thick Au film, which acts as the PEC substrate in our configuration. A further illustration of the fabrication process can be found in the Supplementary Information, Figure S1. The geometrical parameters were optimized to support an anapole resonance when the silicon disks are placed on either a gold film (resonator on mirror, ROM) or a glass substrate (resonator on insulator, ROI) at the wavelength of the optical pump (1550 nm). The electron microscopy images of the fabricated ROMs are shown in Fig. 2a. The multipolar contributions to the scattering far-field for ROMs for different disk radii around the anapole resonance are shown in Fig. 2b, where the Cartesian electric dipole and toroidal dipole contributions are obtained by performing the current Cartesian multipole expansion^33^. C_sca_ is the calculated overall scattering cross section based on the scatterings when the nanodisk is placed on the gold film compared to the field in the absence of the nanodisk^27^. For disk radii near 450 nm (indicated by the gray line), there is a dip in the scattering spectrum accompanied by an enhanced near-field profile inside and around the disk, indicating the anapole configuration. Importantly, the toroidal dipole mode is also resonantly excited and spatially overlaps with the electric dipole mode due to the formed magnetic hotspot at the interface as discussed above. This is a clear advantage compared to the previously studied anapole modes, which were excited by all-dielectric resonators on an insulator^33,34^. As a result, a much stronger near-field profile is achieved in such a configuration (Fig. 2c) compared to the normal anapole resonators (see Supplementary Information, Section II, Figures S2-S4). Based on the multipolar decomposition (see Fig. 2b), the anapole mode is also accompanied by the magnetic quadrupole moment due to an uncompensated circulating magnetic field in a flat geometry, formed by two anti-parallel magnetic dipole moments at the nodes of the poloidal current distribution^33^. Thus, the total scattering in the vicinity of the anapole mode is dominated by the magnetic quadrupole radiation, while electric dipole radiation is completely suppressed.

**Fig. 3:**
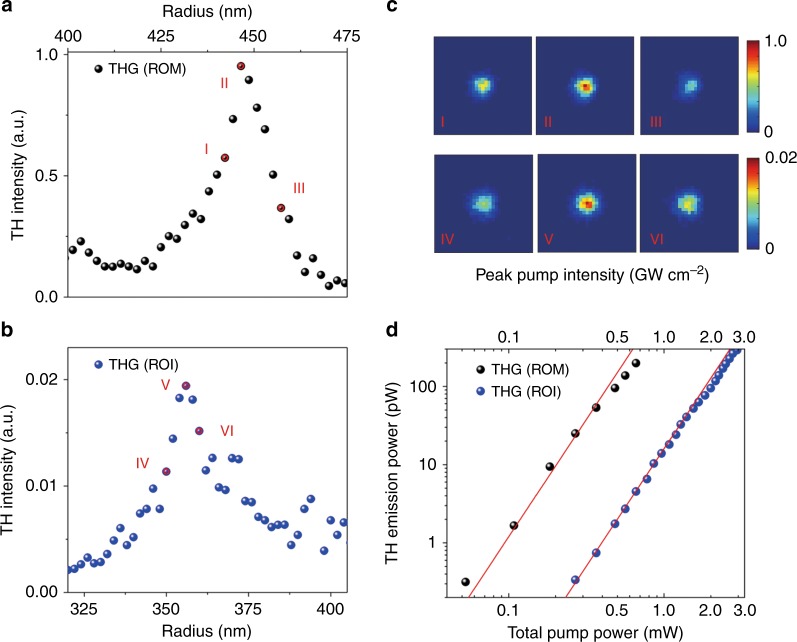
Experimentally measured TH intensity for **a** ROM and **b** ROI configurations for different disk radii around the anapole resonances. **c** The corresponding TH intensity image for several disk radii around the anapole resonances. **d** Measured TH power as a function of pump power, where the cubic dependence is given by the red lines *y* *=* 1200*x*^3^ (ROM) and *y* *=* 16*x*^3^ (ROI). The peak pump intensity was estimated from the average pump power with 1 ps pulses at a 40 MHz repetition rate

Figure 3 shows our experimental results for THG measurements. A picosecond laser beam at the wavelength of 1550 nm was focused by an objective with a numerical aperture of 0.7 onto the nanodisks. Figure 3a, b shows the experimentally measured TH emission intensity in silicon nanodisks around the anapole resonances for both types of configurations (ROM and ROI, respectively). The total power for the input pump beam after the objective is 0.49 mW, leading to the maximum peak intensity value of *I*_0_=0.5 GW cm^−2^. Resonant nonlinear emissions can be clearly observed for ROMs with *r* *=* 450 nm and for ROIs with *r* *=* 355 nm. The measured THG spectra provide an obvious indication of the contributions from the anapole resonance at the fundamental wavelength to the nonlinear response of the structure. The much higher THG emissions from the ROM configuration reflects the ability of our designed nanostructures to boost the nonlinear process. Figure 3c shows the measured TH intensity image taken around the anapole resonance under the ROM and ROI configurations. The high contrast between the disk and surrounding substrate indicates negligible TH emission from the substrate in both cases. The TH intensity follows the cubic trend of the pump power, as shown in Fig. 3d. As can be seen, by using the same pump configuration with a maximum peak intensity value of *I*_0_=0.5 GW cm^−2^, nearly 100 times enhancement of the TH emission under the ROM configuration compared to the TH emission from the ROI configuration was experimentally observed. This result is in line with the theoretical predictions, which show more than 100 times enhancement in the total nonlinear emission (see Supplementary Information, Figure S5).

**Fig. 4:**
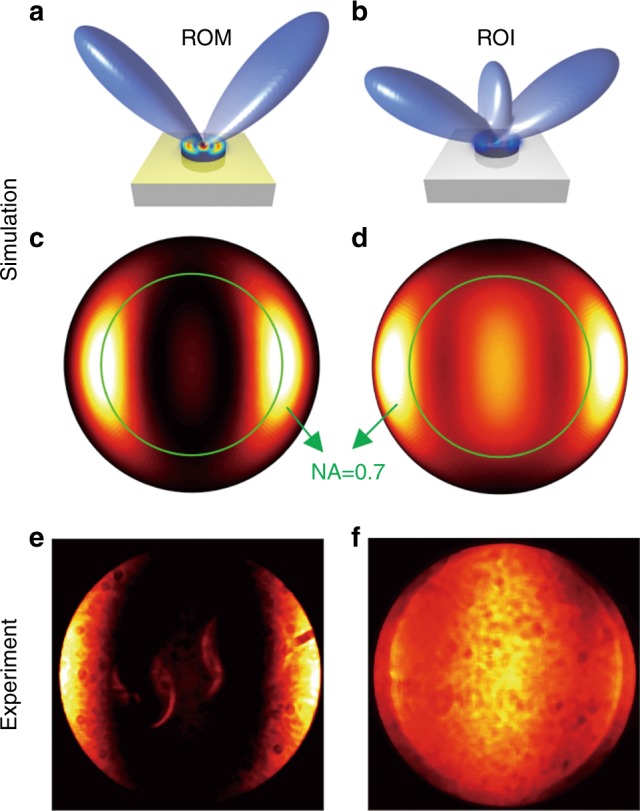
**a**, **b** Calculated backward directionality of TH emission from ROM and ROI configurations, respectively. **c**, **e** Calculated and **d**, **f** measured two-dimensional projections of directionalities, for the numerical aperture of the objective used, from ROM and ROI configurations, respectively. The inner green circles in **c**, **d** indicate the area experimentally accessible by the objective used, corresponding to the measured image in **e**, **f**

We further investigate the nonlinear emission properties by measuring the back-focal plane (BFP) images. A femtosecond laser operating at the wavelength of 1550 nm with a 100 fs pulse width at the repetition rate of 80 MHz was used (see Supplementary Information, Figure S6). By projecting the back-focal plane (Fourier space) images for an objective lens with a numerical aperture of 0.7 onto the camera, we have measured the collected backward TH radiation patterns. We performed numerical simulations in which the calculated TH far-field intensity distribution was projected onto the back-focal plane. As shown in Fig. 4, our experimental and simulated results agree very well. Although for both ROM and ROI configurations, the nonlinear process is driven by the anapole modes at the fundamental wavelength during the linear process, the radiation patterns differ from each other due to the differently generated nonlinear multipoles in these two configurations (see Figures S7 and S8 in the Supplementary Information). Additionally, a halo is observed at the back-focal plane in the ROM configuration due to the reflection of the nonlinear emission from the backside of the glass film.

**Fig. 5:**
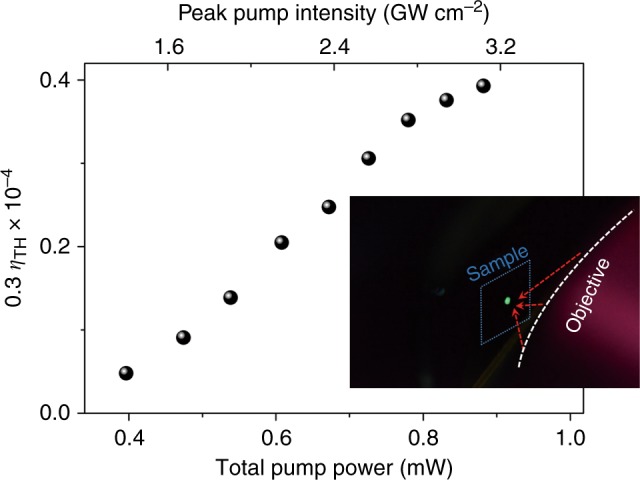
Experimentally measured conversion efficiency of the THG process at the anapole resonance under the ROM configuration. The inset shows a photographic image of the TH emission on the sample. The peak pump intensity was estimated from the average pump power with 100 fs pulses at an 80 MHz repetition rate

We also calibrated the collected TH emissions of the ROM configuration with respect to the pump power. Figure 5 gives the collected TH conversion efficiency, which is defined as the ratio between the collected TH emission power and the input pump power on the disk size (for TH emission power dependence, see Figure S9 in the Supplementary Information). During the measurement, no two-photon absorption or other nonlinear effect was observed for our pump power range (see Figure S10 in the Supplementary Information). A TH conversion efficiency of 0.004% is achieved using a peak pump intensity as low as only 3.0 GW cm^−2^, which is the lowest peak pump intensity reported to achieve such a high efficiency to date. Based on the objective’s numerical aperture employed, only one third of the TH emission is collected through the objective and the camera (see Figure S5 in the Supplementary Information). Therefore, the total conversion efficiency of our ROM configuration is estimated to exceed 0.01%. Importantly, we achieve such exceptional enhancement from a single nanodisk rather than using complex configurations such as lattice structures.

The inset of Fig. 5 shows a photographic image of the sample irradiated with a 1550 nm pump laser as indicated by the red arrow. The TH emission from an individual disk can be clearly observed. It is worth mentioning that observation of the TH light on a sample by the naked eye has been previously reported^22^. However, due to the highly efficient TH response in our ROM configuration, the TH light can be observed by the naked eye not only on the sample but also in the light pathway (see Supplementary Information, Figure S6).

## Discussion

The observed strong THG emission is due to the two advantages of our designed nanostructure: first, in the linear process, significant near-field enhancement is obtained inside the resonator near the anapole state due to the spectral overlap of the resonantly excited Cartesian electric and toroidal dipole moments (as shown in Fig. 2b). This results in a more strongly excited anapole state compared to the previously reported results. Second, the enhanced anapole resonance state at the fundamental wavelength combined with the mirror surface on the nonlinear resonator produces a mirror image of the nonlinear source and thus produces stronger total nonlinear emission (as shown in Figs. 3 and 5). It is important to note that when a pump pulse is incident on an ideal metallic mirror (PEC surface), two times enhancement of the pump pulse is obtained due to the reflected wave from the surface. This condition is broken when a resonator is placed on the metallic surface because the light is scattered by the resonator before reaching the metallic mirror. However, by utilizing the mirror effect on the linear and nonlinear process through our designed nanostructure, a 100 times enhancement of THG emission via the ROM system is obtained both theoretically and experimentally (as shown in Fig. 3 and Figure S5 in the Supplementary Information).

In summary, we have presented a mirror-enhanced anapole system as a novel approach to significantly enhance third harmonic generation. It is shown that by using an anapole resonator on mirror system, one can obtain (i) high near-field enhancement based on an overlap between the resonantly excited Cartesian electric and toroidal dipole moments, which is a property that cannot be achieved by regular anapole resonators, and (ii) free-charge oscillations within the interface that act as an extra nonlinear source below the interface and further increase the total achieved nonlinear emission. Therefore, our configuration can enhance the TH emission by two orders of magnitude compared to a regular anapole resonator on an insulator substrate. This leads to an unprecedented total TH conversion efficiency of 0.01%. Our approach provides a new platform to manipulate and boost the all-dielectric Mie resonators to achieve enhanced nonlinear performance. We believe that our results are an important step towards the use of nonlinear sources at the nanoscale with high efficiencies for real applications such as nanolasers, quantum sources, and nonlinear holograms.

## Materials and methods

### Sample fabrication

1. Silicon disk on gold surface: Amorphous silicon nanodisks with a thickness of 200 nm were fabricated on a Au film with a thickness of 200 nm on a silicon substrate. First, 200 nm of Au was evaporated onto the substrate after evaporating 2 nm Ti to enhance the adhesion. Then, a 200 nm hydrogenated amorphous silicon layer was deposited on the substrate by plasma-enhanced chemical vapor deposition (Oxford PlasmaLab System 100). Subsequently, SiO_2_ nanodisks were fabricated with electron-beam lithography (Raith 150). Using the selective reactive-ion etching process, SiO_2_ disks were transferred to the silicon film. Finally, the residual SiO_2_ disks were removed via wet etching. A schematic illustration is shown in Figure S1 - Supplementary Information.
2. Silicon disk on glass film: Amorphous silicon nanodisks with a thickness of 200 nm were fabricated on a glass substrate with a thickness of 180 µm. First, a 200 nm thick amorphous silicon layer was deposited on the glass substrate by plasma-enhanced chemical vapor deposition (Oxford PlasmaLab System 100). Subsequently, positive electro-resist was spin-coated over the film. We then exposed the resist by applying electron-beam ithography (Raith 150) and development. A 50 nm Cr film was evaporated onto the sample, followed by the lift-off process to generated Cr masks. Using reactive-ion etching processes, Cr disks were transferred to the silicon film. The residual Cr disks were further removed by wet Cr etching.

### Experimental system

THG intensity characterizations were performed with a commercial WiTec alpha300S system, using excitation by a picosecond laser at 1550 nm with 1 ps pulses at a repetition rate of 40 MHz. An objective with NA = 0.7 was used to focus the beam on the sample and collect the TH signal in the backward direction. THG efficiency calibration and back-focal plane image measurement were performed using a home-built optical microscope setup. A femtosecond laser at 1550 nm with a 100 fs pulse duration at an 80 MHz repetition rate (Toptica FemtoFiber laser system) was focused with an NA = 0.7 objective and used to pump the sample. The TH signal was collected by the same objective in the backward direction. A dichroic mirror was used in front of the objective lens to direct the backward TH onto a camera. A pair of confocal lenses were used to build a back-focal plane image of the TH radiation on the camera.

### Numerical methods

The linear and nonlinear responses of our nanodisks were modeled numerically using the finite-element method in COMSOL Multiphysics in the frequency domain. We assumed the undepleted pump field approximation and followed two steps to model the nonlinear response^26,27^. The linear scattering at the fundamental wavelength was simulated, and the nonlinear polarization induced inside the nanodisk was obtained. We then employed the obtained nonlinear polarization as a source for the electromagnetic simulation at the harmonic wavelength to obtain the generated TH field. For amorphous silicon, the nonlinear susceptibility tensor *χ*^(3)^ was considered as a constant scalar value with *χ*^(3)^ = 2.45 × 10^−19^ m^2^ V^−2^ at *λ*≈1550 nm;^53,54^ thus, the induced nonlinear polarization components at the TH wavelength could be simplified as *P*^(3)^ = *ε*_0_ *χ*^(3)^*E*_*i*_ (***E E***), where *ε*_0_ is the vacuum permittivity, and *E*_*i*_ is a component of the electric field ***E***, with *i* referring to the components in the *x*, *y*, and *z* directions.

## Electronic supplementary material

Supplementary Information
