# Supplementary material for: Boosting third-harmonic generation by a mirror-enhanced anapole resonator
Source: Light Sci Appl. 2018 Jul 25;7:44. doi: 10.1038/s41377-018-0051-8 (PMC6107010; doi:10.1038/s41377-018-0051-8)
Supplement: Supplementary file 1 — Supplementary Information [file 41377_2018_51_MOESM1_ESM.docx]

**Supplementary Information:**

**Boosting third-harmonic generation by a mirror-enhanced anapole resonator**

**Lei Xu**1,2,+**, Mohsen Rahmani**1,+**, Khosro Zangeneh Kamali**1**, Aristeidis Lamprianidis**1**, Lavinia Ghirardini**1,3**, J**u¨**rgen Sautter**1,4**, Rocio Camacho-Morales**1**, Haitao Chen**1**, Matthew Parry**1**, Isabelle Staude**4**, Guoquan Zhang**5**, Dragomir Neshev**1,***, and Andrey E. Miroshnichenko**2,*

*1Nonlinear Physics Centre, The Australian National University, Canberra ACT 2601, Australia*

*2School of Engineering and Information Technology, University of New South Wales, Canberra ACT 2600, Australia*

*3Department of Physics, Politecnico di Milano, Piazza Leonardo Da Vinci 32, 20133 Milan, Italy*

*4Institute of Applied Physics, Abbe Center of Photonics, Friedrich Schiller University Jena, Albert-Einstein-Str. 15, 07745 Jena, Germany*

*5The MOE Key Laboratory of Weak Light Nonlinear Photonics, School of Physics and TEDA Applied Physics Institute, Nankai University, Tianjin 300457, China*

*Dragomir.Neshev@anu.edu.au; Andrey.Miroshnichenko@unsw.edu.au

+these authors contribute equally to this work

Contents

1. Nanofabrication
2. Linear Scattering and Near-Field Distributions at the Anapole Resonance
3. Third-Harmonic Generation Calculation
4. Experimental Setup for Nonlinear Measurements
5. Nonlinear Multipolar Generation
6. TH emission power dependence measurement
7. Nonlinear emission spectrum measurement
8. **Nanofabrication**

Figure S1 gives the schematic illustration of the fabrication process for our sample.


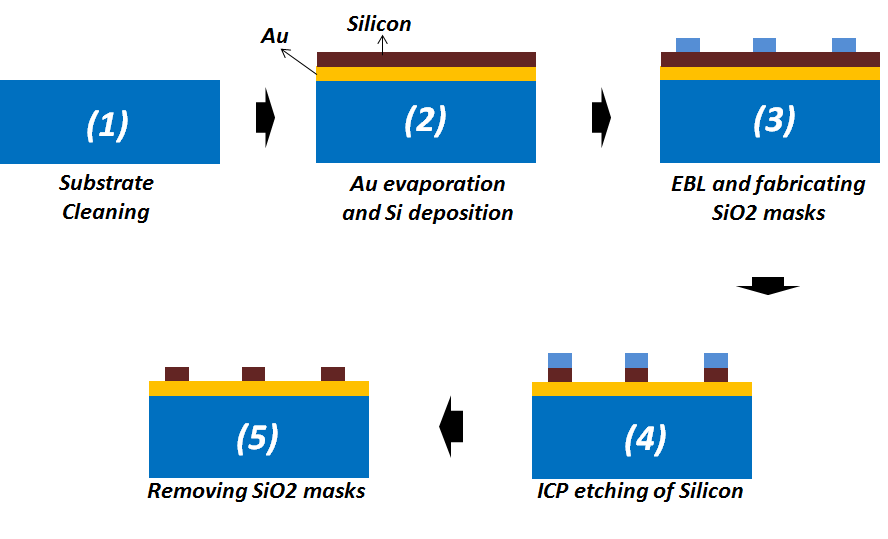


Figure S1. Illustration of the nanofabrication steps.

1. **Linear Scattering and Near-Field Distributions at the Anapole Resonance**

Here, we provide the linear scattering for ROM and ROI configurations around their anapole resonances, respectively, as shown in Figure S2(a) and S2(b). The excitation of Cartesian electric and toroidal dipole moments are shown in Figure S2(c-f). It worth noting that due to the presence of the substrate, the scattering magnitude of Cartesian ED and TD modes are usually not equal, while they are very close to each other.


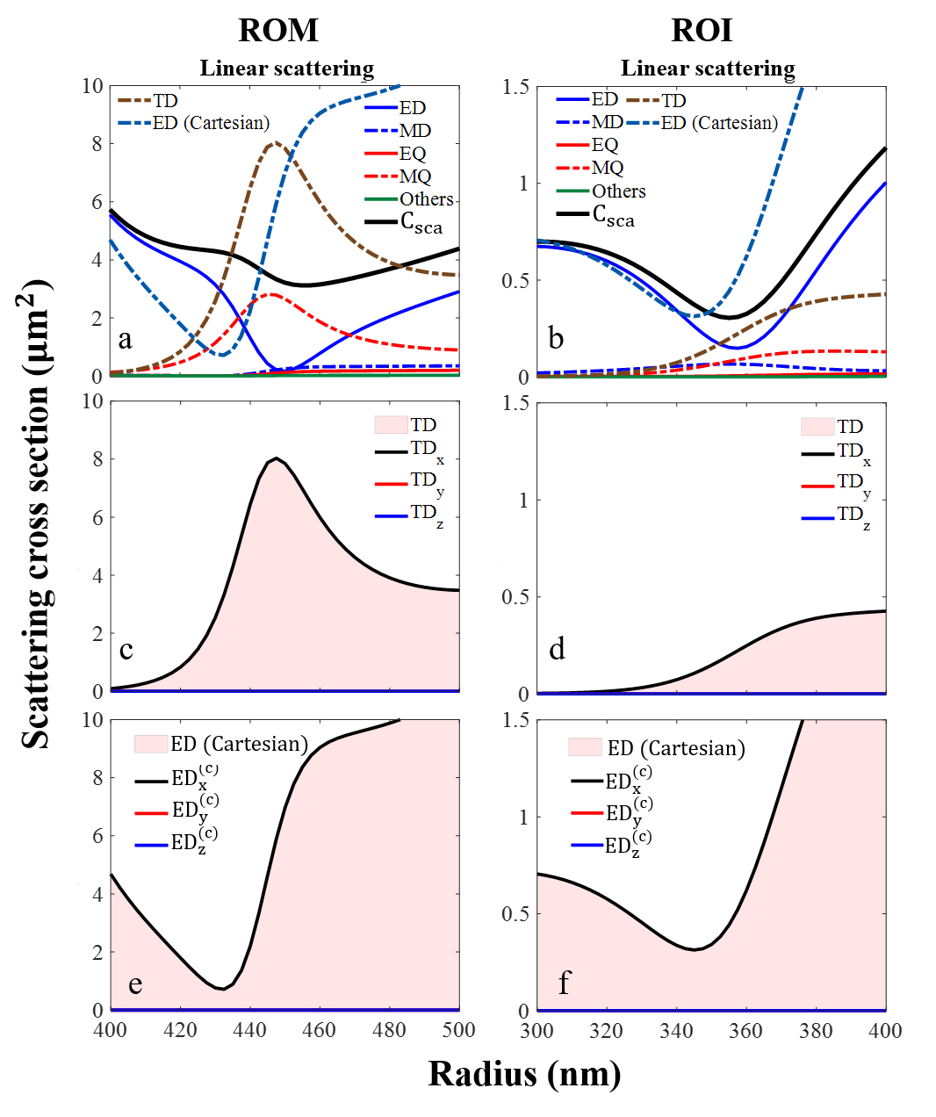


Figure S2. (a,b) Calculated linear scattering and multipolar decomposition for ROM and ROI configurations with different disk radii by plane wave excitation. (c,d) The excitation of toroidal dipole moments for ROM and ROI configurations, respectively. (e,f) The excitation of Cartesian electric dipole moments for ROM and ROI configurations, respectively.

Figure S3 gives the electric near-field distributions for ROM and ROI configurations at the anapole resonance. The magnetic near-field distributions for ROM and ROI configurations are shown in Figure S4. A magnetic hotspot is formed near the interface between disk and gold film for the ROM configuration.


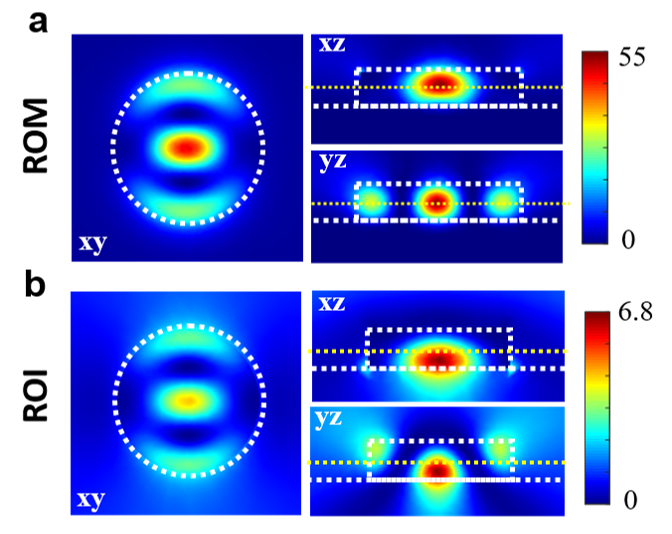


Figure S3. The spatial profile of the electric field $\mathbf{I/}\mathbf{I}_{\mathbf{0}}$ from xy, yz, xz cross sections for (a) ROM and (b) ROI configurations at the anapole resonance.


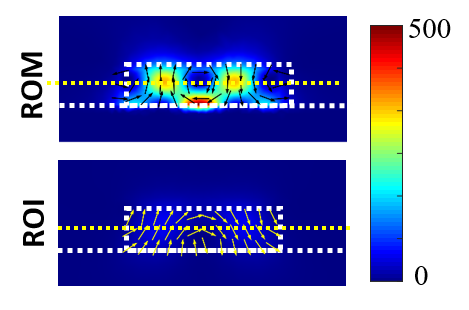


Figure S4. The spatial profile of the magnetic field${\boldsymbol{|}\mathbf{H}\boldsymbol{|}}^{\boldsymbol{2}}\mathbf{/}{\mathbf{H}_{\mathbf{0}}}^{\boldsymbol{2}}$ from xz cross sections for ROM and ROI configurations at anapole resonance. The arrows indicate the magnetic vector fields.

1. **Third-Harmonic Generation Calculation**

For comparison with our experimental results, we calculate the total third-harmonic emission and the collected third-harmonic emission, taking into account the NA of the objective used in experiment, for both ROM and ROI configurations, respectively, as shown in Figure S5. The peak pump intensity is set to $I_{0}=1.0 GW/\mathrm{cm}^{2}$ in our simulation. As can be seen, the total nonlinear emission is more than 100 times higher from ROM configuration as compared to the ROI configuration. For both cases around the anapole resonance, only one third of the backward nonlinear emission will be collected by the objective and the detector.


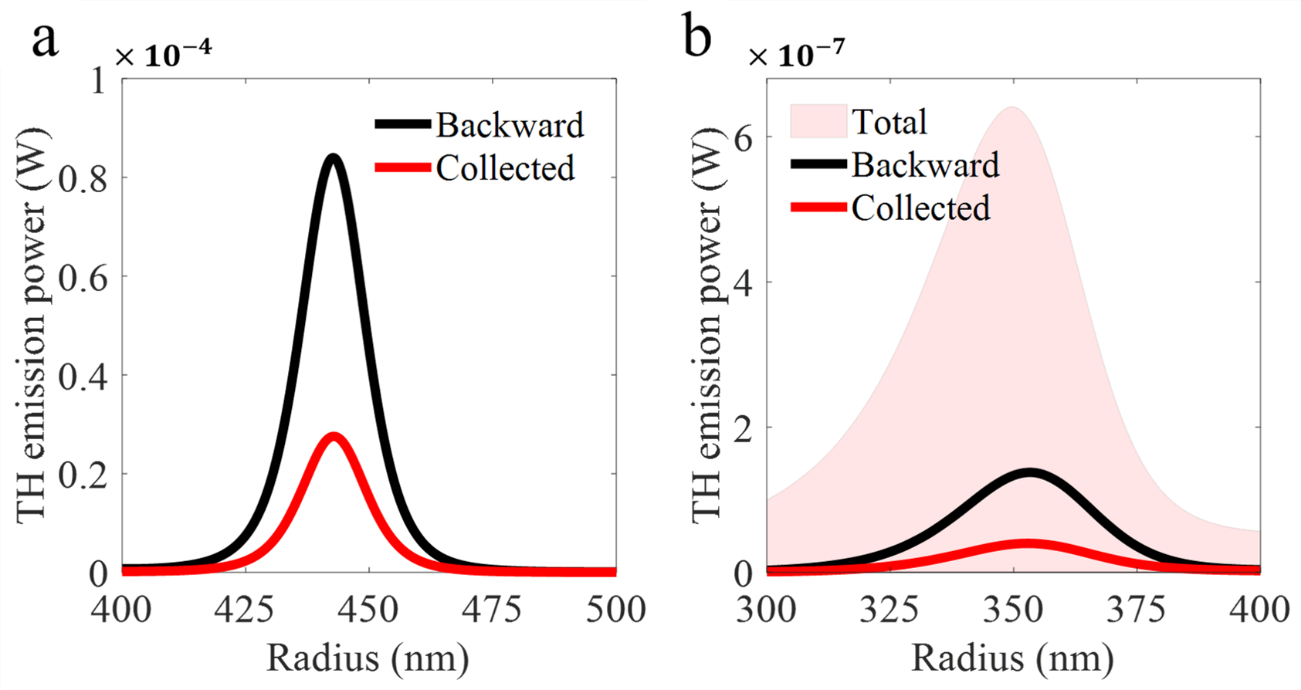


Figure S5. Calculated total TH emission power (dark curve) and collected TH emission power by Objective (red curve) for ROM (a) and ROI (b) nanosystems, respectively.

1. **Experimental Setup for the Nonlinear Measurements**

The schematic of the experimental setup for nonlinear measurements and observations of the BFP image is shown below. It is worth noting that we use different laser for the THG intensity mapping measurement (Figure 3), and the THG efficiency calibration and back-focal plane image measurements due to the restriction that the Toptica FemtoFiber laser system (femtosecond laser at 1550 nm with 100 fs pulse duration at 80 MHz repetition rate) cannot be integrated into the commercial WiTec alpha300S system.


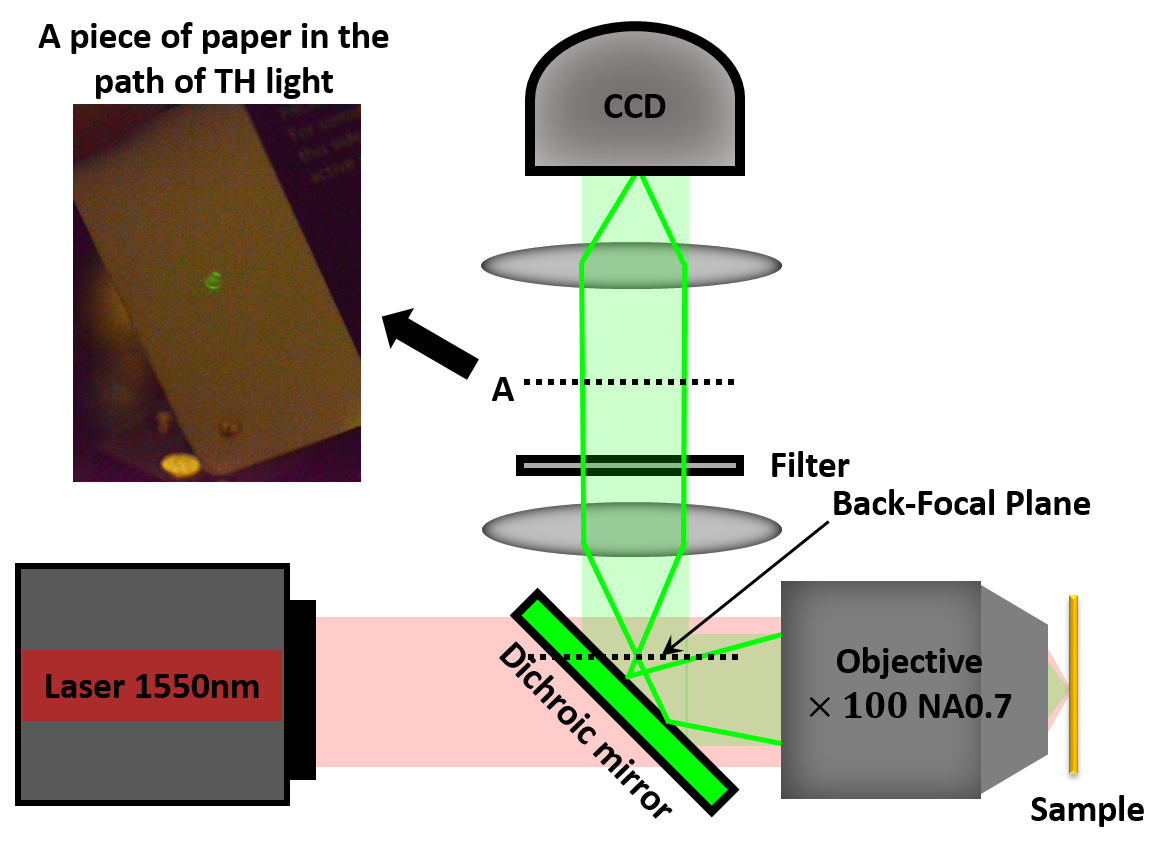


Figure S6. Schematic of the experimental setup for the nonlinear measurements. The inset shows a photographic image of the obtained TH emission by putting a block in the path of the light at position “A”.

1. **Nonlinear Multipolar Generation**

We further investigate the nonlinear emission properties by performing nonlinear multipolar decompositions. Figures S7 and S8 give the nonlinear multipolar generations under ROM configuration and ROI configuration, respectively, and the calculated near-field profile at the corresponding resonance positions. Different nonlinearly generated multipoles are observed from the anapole resonators between these two configurations. This is due to the fact that under ROM configuration, the effect of mirror on the nonlinear source is equivalent to introducing another image of a nonlinear source and the pump beneath the interface. The interference and coupling between the nonlinear source and its image counterpart will further affect the nonlinearly generated multipoles and thus the far field patterns, in addition to its contribution to the nonlinear emission enhancement.


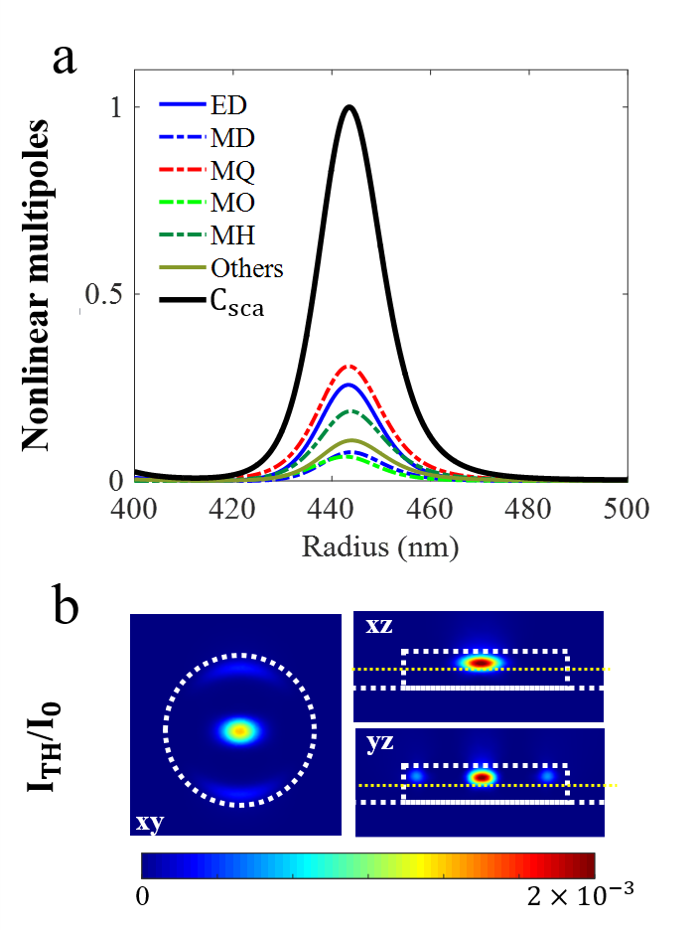


Figure S7. (a) Nonlinear multipolar generation as a function of the disk radius under ROM configurations, and (b) the near-field profile at the anapole resonance. The input pump intensity is I_0_ =1.0 GW/cm^2^ in this simulation.


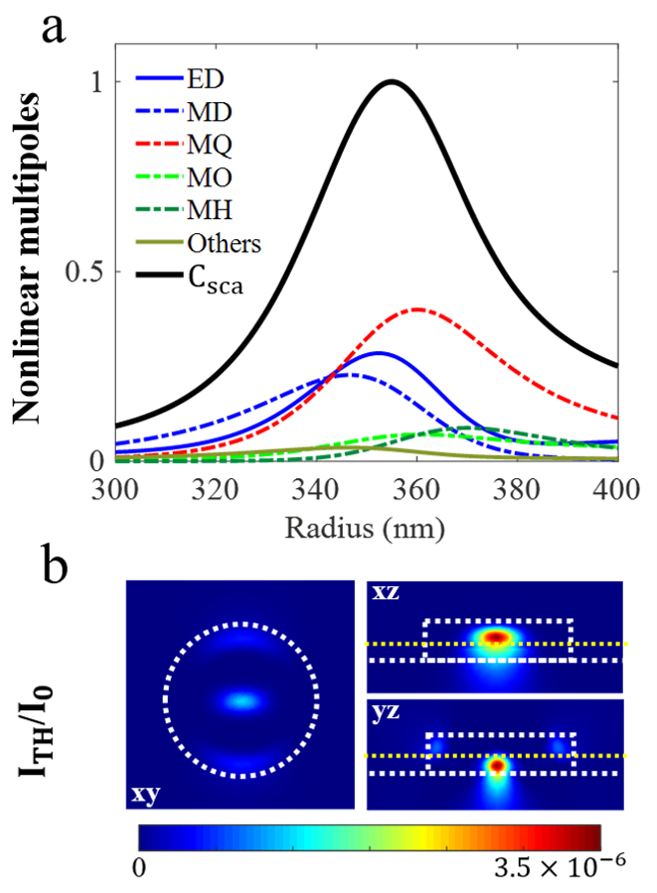


Figure S8. (a) Nonlinear multipolar generation as a function of the disk radius under ROI configurations, and (b) the near- field profile at the anapole resonance. The input pump intensity is I_0_ =1.0 GW/cm^2^ in this simulation.

A significant local THG near-field enhancement can clearly be seen from the ROM configuration as compared with ROI configuration, in agreement with the measured results shown in Figure 3 in the main text. Thus showing a greatly enhanced TH emission under ROM configuration.

1. **TH emission power dependence measurement**

The dependence of TH emission power on the total input pump power are shown in Figure S9. The TH emission starts to saturate around a pump power of 0.85 mW, corresponding to a peak pump intensity around 3$GW/\mathrm{cm}^{2}$ (shaded area in the figure).


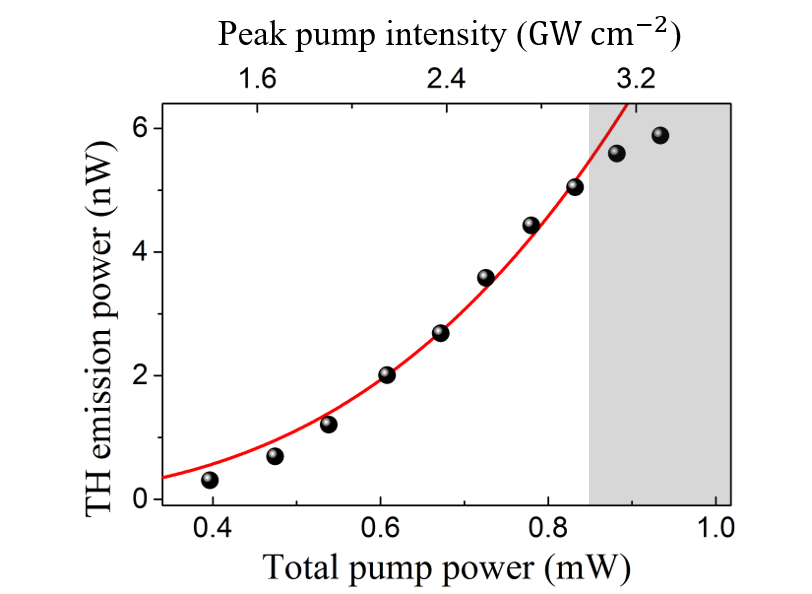


Figure S9. The measured TH emission power dependence on total input pump power for anapole resonator under ROM configuration

1. **Nonlinear emission spectrum measurement**

Figure S10 shows the measured emission spectrum from anapole resonator under ROM configuration in the visible/near-infrared range at a high pump peak intensity around 3$GW/\mathrm{cm}^{2}$. As can be seen, no two-photon absorption and other nonlinear effect was observed for our pump power range, only TH emission is observed in our experiment.


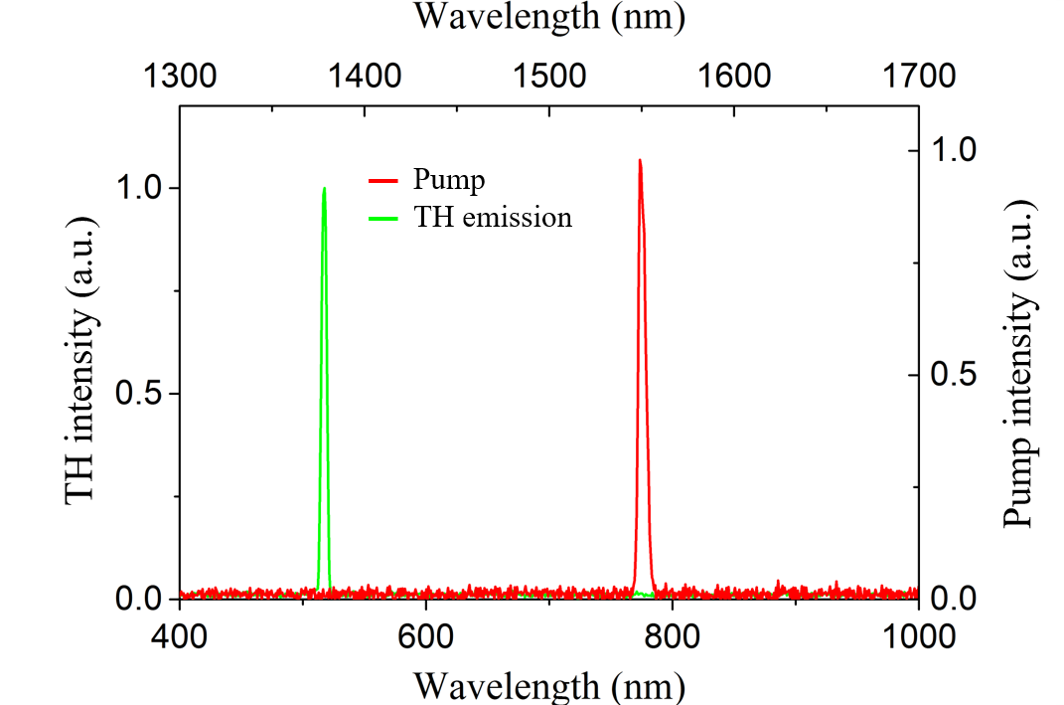


Figure S10. The measured TH emission spectrum (green curve) from anapole resonator under ROM configuration in the visible/near-infrared range at a high total input pump power 0.8 mW, corresponding to a peak intensity around 3$GW/\mathrm{cm}^{2}$. The red curve shows the spectrum of the pump laser.
